# Short‐term performance responses of an intertidal fish to sedimentation and warming

**DOI:** 10.1111/jfb.70494

**Published:** 2026-05-08

**Authors:** Anna Carolina Resende, Lucy Campbell, Catarina Vinagre, Alice Rogers

**Affiliations:** ^1^ School of Biological Sciences Victoria University of Wellington New Zealand Wellington New Zealand; ^2^ CCMAR – Centre of Marine Sciences University of Algarve Faro Portugal; ^3^ Universidade do Algarve Faro Portugal; ^4^ MARE – Marine and Environmental Sciences Centre Universidade de Lisboa, Faculdade de Ciências Lisbon Portugal

**Keywords:** acute stress, foraging, multistressor, New Zealand, swimming speed

## Abstract

Climate change is altering coastal ecosystems by causing environmental fluctuations, such as increases in temperature and turbidity, which pose major implications for fish physiology and behaviour. Increases in temperature affect fish food intake, swimming capacity and oxygen delivery, while increases in turbidity can impair or enhance prey detection, as well as affect fish movement. Since these stressors often act together, understanding their combined effects is critical. We investigated how short‐term increases in temperature and turbidity, both separately and in combination, influenced the foraging and swimming performance of the common triplefin, *Forsterygion lapillum* Hardy 1989, and measured oxygen consumption during acute thermal ramping to explore links between thermal tolerance and performance. Results show that *F. lapillum* strike speed was slower in high turbidity treatments, indicating that *F. lapillum* relies on visual cues to feed, and consequently, fish foraging performance is impaired in sedimented waters. Moreover, fish routine swimming speed and burst speed were unaffected by any treatment, suggesting that *F. lapillum* can adapt its swimming performance to the increases in temperature and turbidity that were tested. During acute thermal ramping, fish oxygen consumption rate was found to increase only at temperatures above 24°C. This can explain *F. lapillum*'s lack of adjustment in swimming speed, burst speed and strike speed to increases in temperature during the experiment and suggests that oxygen delivery starts to be compromised at temperatures above this threshold. Our findings highlight *F. lapillum* resilience to moderate temperature increases but reveal vulnerability to increased sedimentation due to reduced foraging efficiency.

## 1 INTRODUCTION

Climate change poses a major threat to marine ecosystems (Guo et al., 2022; Hoegh‐Guldberg & Bruno, 2010; Tittensor et al., 2021). It is expected to drive chemical and physical changes in coastal environments, including rising temperatures (Guo et al., 2022) and increased turbidity (IPCC, 2023; Roberts, 2012). Such altered conditions can influence the behaviour and survival of fish (Kreiling et al., 2021; Perry et al., 2005; Scott et al., 2019).

Temperature is an important abiotic factor that shapes fish behaviour, and increases in temperature can affect fish food intake (Salin et al., 2016; Scott et al., 2017) and swimming capabilities (Faria & Gonçalves, 2010; Zhou et al., 2019). Previous studies have shown that, for many fish species, moderate temperature increases lead to increased foraging behaviour due to higher metabolic rate (Ahmad et al., 2014; Brett, 1971; Volkoff & Rønnestad, 2020). This pattern is consistent with the thermal performance curve (TPC) framework, which describes how species performance varies with temperature (Huey & Stevenson, 1979; Nati et al., 2016). Performance typically peaks within an optimal temperature range and declines outside this range, approaching zero near critical thermal limits (Angilletta, 2006; da Silva et al., 2019; Schulte et al., 2011). Accordingly, as temperatures exceed a species’ optimal range, fish feeding performance may decline prior to reaching CTmax, supporting the use of CTmax as an indicator of upper thermal limits (Volkoff & Rønnestad, 2020).

Temperature can influence fish locomotor performance and predation success, thereby affecting their ability to detect and capture prey (Killen et al., 2013; Volkoff & Rønnestad, 2020). Consequently, feeding performance is closely linked to swimming performance. Swimming performance is a key determinant of survival in fish, not only because it underpins successful prey capture, but also effective predator avoidance (Pang et al., 2011; Zhou et al., 2019). Fish can either be more active and swim faster when facing increases in temperature (Allibhai et al., 2023; da Silva et al., 2019; James, 2013) or be less active and decrease movement due to limited oxygen delivery (Pörtner, 2010; Zhou et al., 2019). Thus, the investigation of a species oxygen consumption during acute thermal ramping can provide insight into how metabolic demand changes with temperature and help contextualize potential links to swimming performance (Volkoff & Rønnestad, 2020, Zhou et al., 2019). Turbidity is another environmental variable that affects fish behaviour and performance (Bass et al., 2023; Seers & Shears, 2015). Increases in turbidity in coastal environments occur through the input or resuspension of fine sediment particles and other dissolved organic matter (Seers & Shears, 2015; Smith & Schindler, 2009; Zanghi & Ioannou, 2025). In highly turbid ecosystems, the ability of both predators and prey to detect each other is impaired (Utne‐Palm, 2002). Previous studies found that increased turbidity can interfere with prey capture by either: (a) increasing the contrast of a prey against its background, facilitating prey detection (Hinshaw, 1985; Wing et al., 2021) or (b) a reduction in the visual field, impairing the predator's ability to detect prey (Hess et al., 2017; Wenger et al., 2012; Wenger et al., 2018), ultimately altering fish foraging performance. Similarly, fish swimming performance can be altered under high turbidity, where fish can either: (a) be more active and increase movement (Johannesen et al., 2012; Wishingrad et al., 2015); or (b) be less active and reduce movement, potentially making them more vulnerable to predation (Allibhai et al., 2023; Kimbell & Morrell, 2015).

Although it is important to understand how temperature and turbidity individually affect fish, it may be unrealistic, as performance is often influenced by their combined effects (McArley et al., 2017; Rosewarne et al., 2016). In intertidal coastal environments, increases in temperature and turbidity frequently co‐occur, exposing resident fish to multiple stressors (Resende et al., 2026). Nearshore shallow areas are characterized by pronounced thermal variability, often resulting in rapid and extreme temperature fluctuations that increase their susceptibility to marine heatwaves (Cook et al., 2022). Fish inhabiting these systems also experience periodic sediment resuspension events and pulses of elevated turbidity (Berman, 2010; Carter & Lewis, 1995). Together, these co‐occurring stressors create a highly dynamic environment with significant implications for fish physiology and performance (Resende, Vinagre, & Rogers, 2025). Moreover, since extreme events are increasing in frequency and duration due to climate change (Guo et al., 2022; IPCC, 2023; Stillman et al., 2025), understanding how fish respond to short‐term changes in environmental variables is essential (McArley et al., 2017; Resende et al., 2022).

The common triplefin, *Forsterygion lapillum* Hardy 1989, is an abundant benthic fish found in intertidal zones throughout New Zealand (McDermott & Shima, 2006). It is primarily an opportunistic carnivore, feeding on small benthic macroinvertebrates such as amphipods, isopods and polychaetes (Resende et al., 2026). The intertidal zone is known to experience daily temperature and turbidity fluctuations due to changing tides, solar radiation and wind (Helmuth et al., 2006; Stillman et al., 2025). Intertidal species usually have the capacity to adapt to these environmental fluctuations by adjusting their behaviour (Gunderson et al., 2019; Stillman et al., 2025). *Forsterygion lapillum* inhabits a variety of heterogeneous environments and can tolerate a wide range of temperatures (McArley et al., 2018; McDermott & Shima, 2006). It is therefore an ideal model species for investigating behavioural responses to environmental stress (Hilton et al., 2010; Khan et al., 2014). Given that turbidity could impact the visual abilities of triplefins, and temperature could impact their metabolism and activity, in this study we aimed to: (a) examine how short‐term increases in temperature and turbidity impact *F. lapillum* foraging and swimming performance, both independently and in combination and (b) investigate *F. lapillum* oxygen consumption during acute thermal ramping to explore its potential associations with fish performance.

## 2 METHODS

### 2.1 Ethics statement

All fish collections and experimental designs were made in compliance with the special permit 711‐4 issued by the Ministry for Primary Industries of New Zealand, and the project was approved by the Animal Ethics Committee of Victoria, Wellington University, application number 0000030063.

### 2.2 Fish collection and maintenance

**FIGURE 1:**
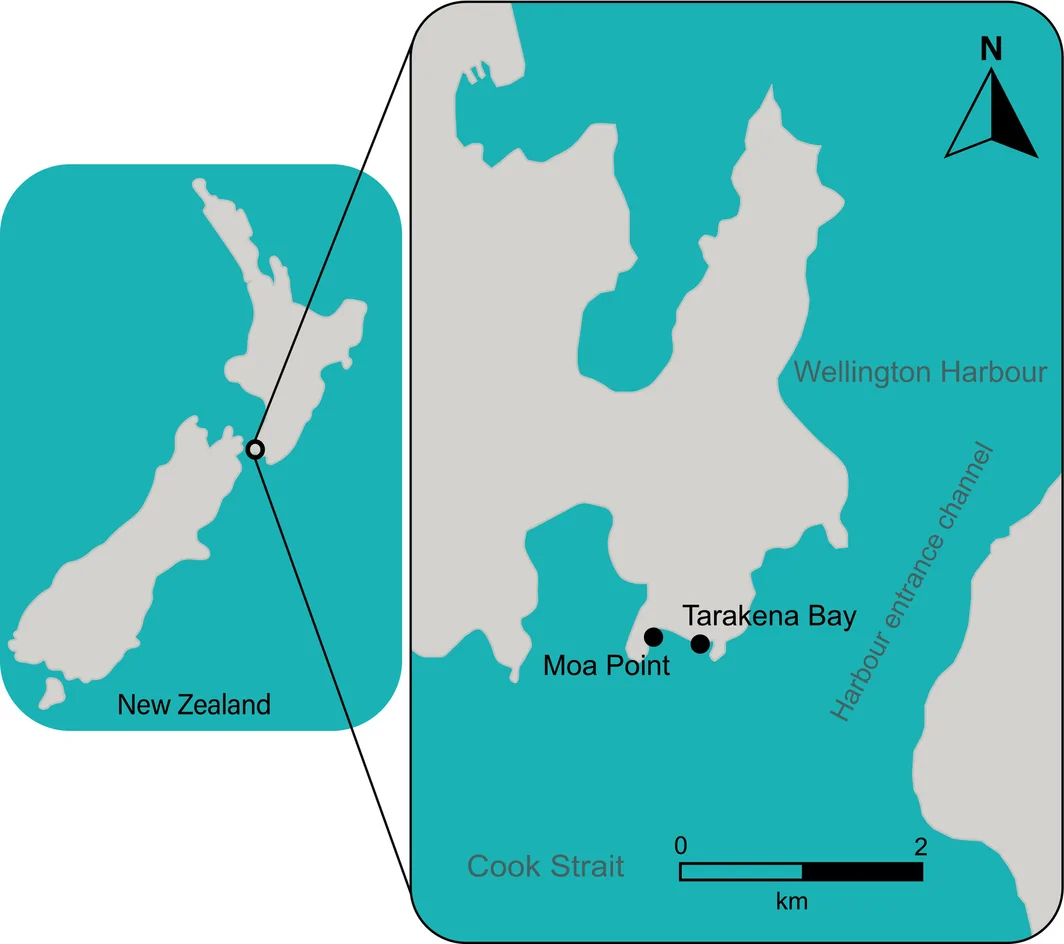
Map showing the location of sampling sites.

*Forsterygion lapillum* (*n* = 56) were collected using hand nets while snorkelling at about 1.5 m depth from neighbouring rocky intertidal habitats with similar conditions, in Tarakena Bay (41°20′38.6″ S, 174°49′8.5″ E) and Moa Point (41°20′26.6″ S, 174°48′38.6″ E) on the Wellington South Coast in November 2024 (Figure 1).

The Wellington South Coast, located within the Cook Strait, is an exposed coastal environment characterized by large swells from southerly winds. Monthly mean temperatures range between 10.9°C and 17.6°C, reaching a maximum of 19.4°C in summer and a minimum of 10.2°C in winter (Supplementary Material Figure S1). Due to the high‐energy characteristics of tidal currents, the bottom substrate of intertidal areas in the Wellington South Coast is in consistent motion, subjected to periodic sediment resuspension, with turbidity ranging from 0 to ~200 nephelometric turbidity units (NTU) (Supplementary Material Figure S2).

After being captured, the fish were transported to the Victoria University Coastal Ecology Laboratory (VUCEL) and placed into an open‐flow 100 L tank with filtered seawater for a minimum of 7 days to acclimatize. Temperature was maintained at 16.3 ± 0.5°C, corresponding to the conditions at the sampling site at the time of collection, and the fish were fed ad libitum, daily with frozen mysis shrimp (AquaOne).

### 2.3 Experiment design

After acclimation, fish were randomly assigned to one of four treatments: (a) control: acclimation temperature and no added sediments (b) Heat stress: increasing temperature with no added sediments (c) Sedimentation stress: acclimation temperature and added sediment and (d) Multi‐stressor: increased temperature and added sediment.

Each fish was placed in its own 50 L tank, where water temperature was controlled by an aquarium controller system (Apex Classic, Neptune Systems), connected to aquarium heaters (220–240 V, Eheims) and two chillers (HC‐500A, Hailea). There were 14 individual replicate trials for each of the four treatments. Water temperature was checked every 20 min with a YSI Pro30 probe. Given that sediment resuspension decreases water clarity, measures of turbidity were used to determine and control sediment levels in treatments with added sediment. Levels were measured once in every trial in each tank with a RBRconcerto3 C.T.D, and reported in nephelometric turbidity units (NTU). Centimetre‐squared graph paper was placed under experimental tanks as a distance reference for video calibration, and fish swimming and behaviour was recorded using a GoPro Hero camera above the tank, oriented at 90°. Horizontal fish movements were recorded and measured along the bottom of the tank. Vertical movements were not measured since *F. lapillum* is demersal and moves predominantly along the seafloor. Fish were starved for at least 12 h prior to experimental trials. Additional details relating to treatment conditions and measurements are described below.

### 2.4 Experimental conditions

**FIGURE 2:**
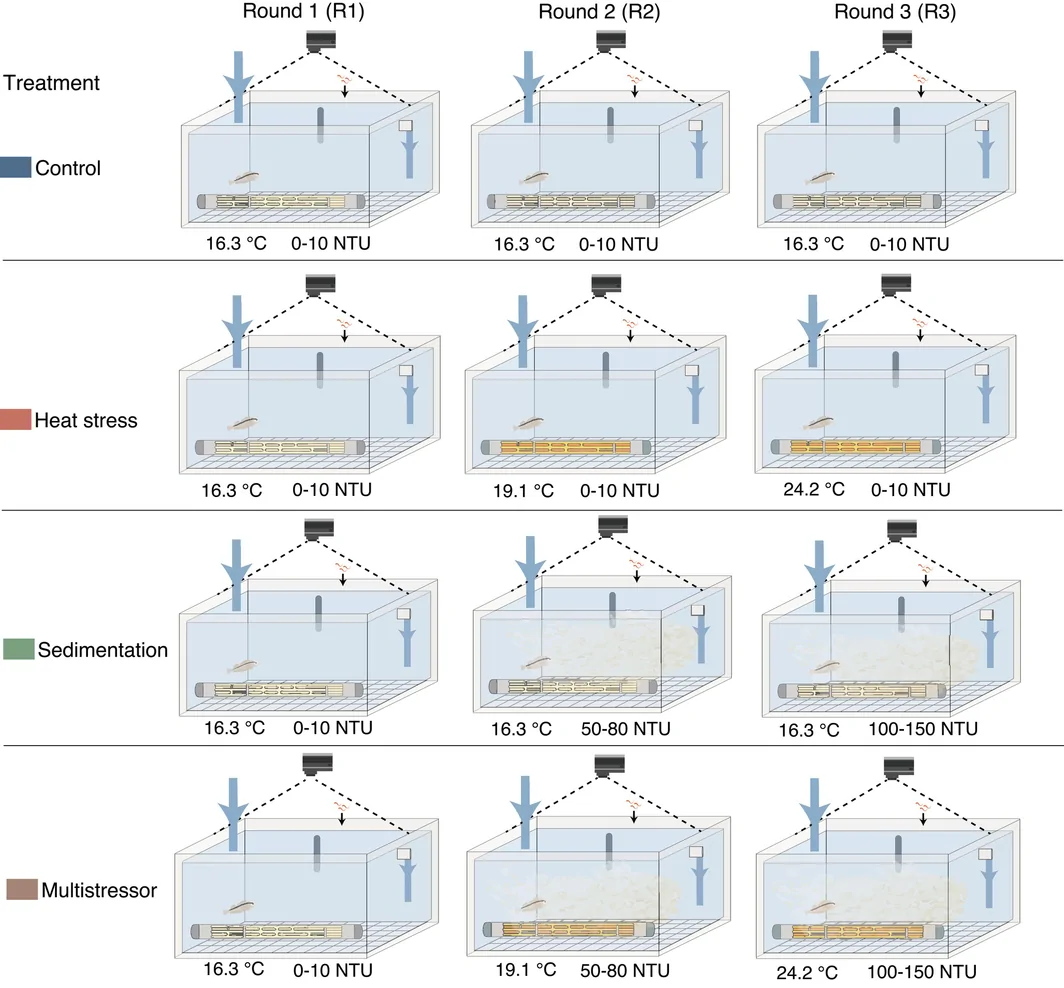
Diagram representing the experimental setup of the present study. Blue arrows indicate water inflow and outflow. Dotted lines indicate the area captured within the GoPro field of view. Small black arrows indicate the approximate positions at which prey were introduced into each tank. Seawater incoming from the Wellington South Coast was first conditioned in a header tank where the seawater temperature was maintained constant with the help of two chillers. The water temperature in the tank was constantly controlled by temperature probes (represented as black rectangles on each tank) connected to the Apex system that would turn on the aquarium heaters (illustrated at the bottom of each tank) in tanks where the temperature needed to be increased. Temperature and turbidity for each treatment in each round are shown in the line beneath each tank. NTU, nephelometric turbidity units.

Treatments were conducted in three 1‐h rounds (R1–R3), where conditions varied depending on the treatment applied, allowing each individual to serve as its own control and enabling comparisons of physiological responses to increasing stressors relative to baseline conditions. For the control, both temperature (16.3 ± 0.5°C) and sedimentation (0–10 NTU) were held constant across all three 1‐h rounds. For the heat stress treatment, acclimation temperature (16.3 ± 0.5°C) was maintained for the first hour (R1), then increased to 19.1 ± 0.4°C in the second hour (R2), and further to 24.2 ± 0.3°C for the final hour (R3). Turbidity was maintained at 0–10 NTU for all three rounds. For the sedimentation stress treatment, turbidity was kept at 0–10 NTU for the first hour (R1). In the second hour (R2), 5 g of sediment was added every 10 min to maintain a turbidity of 50–80 NTU. In the final hour (R3), 5 g of sediment continued to be added every 10 min, increasing the turbidity to around 100–150 NTU. Temperature was maintained at the acclimation of 16.3 ± 0.5°C throughout. For the multistressor treatment, the acclimation temperature (16.3 ± 0.5°C) was maintained for the first hour (R1), then increased to 19.1 ± 0.4°C in the second hour (R2) whilst simultaneously adding 5 g of sediment every 10 min to reach a turbidity of 50–80 NTU. In the final hour (R3), temperature was increased further to 24.2 ± 0.3°C and 5 g of sediment was continually added every 10 min to reach a maximum turbidity of 100–150 NTU (Figure 2). Maximum turbidity and temperature values used in this study were selected based on the extreme conditions recorded along the Wellington South Coast (Supplementary Material Figures S1 and S2).

### 2.5 Performance assessment

*F. lapillum* performance assessments were conducted at the midpoint of each round. Foraging performance was assessed as strike speed, calculated using the distance (in centimetres) between the fish eye and the food, divided by the time in seconds from when food was added to the tank to when the food was captured (modified from Ishikawa et al., 2022). During feeding trials, each fish was provided with 3 bloodworms (AquaOne) that were introduced to the opposite end of the tank, always at the same distance from the fish's eye, using a Pasteur pipette.

Swimming performance was assessed by measuring routine swimming speed (*U*_*rout*_) and burst swimming speed. Routine swimming speed (*U*_*rout*_) was calculated as the total distance (in centimetres) covered by each fish in 120 s of recorded video (based on Faria & Gonçalves, 2010). Routine swimming speed was chosen because it is generally considered to be important for foraging (Faria & Gonçalves, 2010; Fisher & Leis, 2010) and it appears to be more flexible and responsive to short‐term changes (Moyano et al., 2016). Burst swimming speed was assessed as the maximum instantaneous speed of fish after being gently tapped on the tail with a metal rod when they were motionless on the bottom of the tank (da Silva et al., 2019). A minimum of three burst responses were recorded for each fish, and only swimming responses where the fish exhibited a C‐start response were analysed. A C‐start is an escape response characterized by the C shape of the axis of the fish body during initial movement, which is followed by an acceleration to evade the stimulus (Eaton & Emberley, 1991; Wang et al., 2022). All video analyses were performed using the Tracker Video Analysis and Modelling Tool software (Brown, 2024).

### 2.6 Acute thermal ramping and CTmax

Once the performance assessments were finished, fish (*n* = 35) were randomly selected and allowed to rest for a week in the previous acclimation tank. Thereafter, to determine the CTMax, the fish were subjected to acute thermal ramping. Beginning from the acclimation temperature, the fish were exposed to a constant rate of water‐temperature increase of 2°C h^−1^, a rate that is deemed appropriate to determine the acute thermal performance of an intertidal fish (da Silva et al., 2019; Schulte et al., 2011). Individuals were placed in custom‐built respirometry chambers (152 mL volume) and maintained in a thermally controlled water bath where they were left to acclimate for 20 min in the dark (which was a sufficient period to acclimate fish in the respirometry chambers according to preliminary trials, Supplementary Table S1). Oxygen consumption (*MO*_*2*_) of *F. lapillum* was measured during the acute thermal ramping using intermittent‐flow respirometry (Steffensen et al., 1984; Svendsen et al., 2016). In open‐flow states, flush pumps supplied each chamber with aerated, UV‐filtered seawater from the surrounding water bath for 10 min, thus preventing oxygen levels from falling below 90% air saturation. Oxygen concentration (mg O_2_ kg^−1^ h^−1^) inside the chambers was measured every 2 min for 8 min during closed‐flow states at each degree (one round of measurements per hour) increase using a Fibox 4 oxygen probe for a 6‐h period per fish. To record possible bacterial respiration in seawater, one blank measurement was included for each trial (Hess et al., 2017; Strano et al., 2022). MO_2raw_ (mg O_2_ kg^−1^ h^−1^) was calculated using the following equation (Grimmelpont et al., 2023):

(1) $${\mathrm{MO}}_{2\mathrm{raw}}=\left[\left(\frac{∆\left[O_{2}\right]}{∆t}\right)-\left(\frac{∆{\left[O_{2}\right]}_{\text{bact}}}{∆t}\right)\right]\times \frac{V_{\text{resp}}}{m}$$

where *Δ*[O_2_]/*Δ*t (mg O_2_ L^−1^ h^−1^) is the oxygen concentration decrease in the respirometer over time during each MO_*2*_ measurement period and *Δ*[O_2_]_bact_/*Δ*t (mg O_2_ L^−1^ h^−1^) is the oxygen concentration decrease in the blank respirometer. Slopes were obtained through a linear regression, and only slopes with a regression coefficient above 0.95 were considered for further data analysis. *V*_*resp*_ is the respirometry chamber water volume (0.152 L) minus the volume of the fish (considered equivalent to their weight in kg), and m (kg) is the fish body mass.

Since fish respiration is nearly proportional to fish body mass, an allometric correction was done to assess *MO*_*2*_ for a 1 g standard fish:

(2) $${\mathrm{MO}}_{2}={\mathrm{MO}}_{2\mathrm{raw}}\times {\left(\frac{m}{m_{\text{corr}}}\right)}^{1-A}$$

where MO_2_raw (mg O_2_ kg^−1^ h^−1^) is the calculated oxygen consumption with Equation (1), for a fish with mass m (kg) and A is the allometric exponent of 0.89, a universal scaling relationship for fish and metabolic rate suggested by Jerde et al. 2019, resulting in *MO*_*2*_ (mg O_2_ kg^−1^ h^−1^) as the oxygen consumption for a fish with a standard mass, mcorr = 0.001 kg.

Fish were observed continuously, and the value of CTmax was recorded as the non‐lethal endpoint temperature at which the fish showed a loss of body equilibrium (Lutterschmidt & Hutchison, 1997; Borowiec et al., 2024).

### 2.7 Statistical analysis

All statistical analyses were performed in R version 4.4.1, 2024, The R Foundation for Statistical Computing R Core Team (2024). For the performance assessment experiment, two‐way ANOVA tests were performed interacting the independent variables of rounds and treatment to investigate *F. lapillum* performance, with the response variable of strike speed for feeding performance, and routine swimming speed (*U*_*rout*_) and burst swimming speed for swimming performance. To assess changes in *F. lapillum* oxygen consumption during the acute thermal ramping trial, an ANOVA test was used. For significant results, a post hoc pair‐wise test was performed with the *emmeans* package, using the Tukey method (Lenth, 2024). Strike speed, routine swimming speed (*U*_*rout*_) and burst swimming speed are displayed as box plots and MO_2_ is shown as mean and standard error as a line plot, all created using the *ggplot2* package (Wickham, 2016).

## 3 RESULTS

**FIGURE 3:**
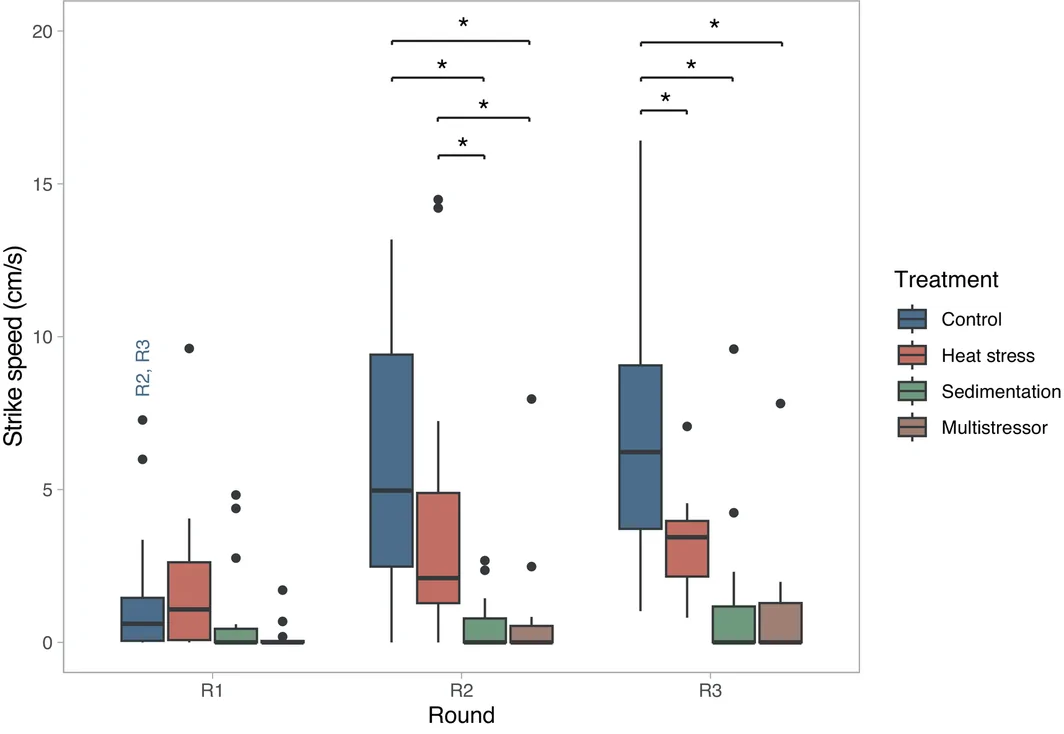
Box plots displaying strike speed of *F. lapillum* over three rounds of the experiment, comparing the four treatments. Asterisks indicate significant differences between treatments per round, with round numbers above the box plots representing differences between rounds within the same treatment.

The foraging performance of *F. lapillum*, assessed by strike speed, significantly changed across rounds and between treatments (ANOVA, F_[2.16]_ = 2.450, *p* = 0.027) (Figure 3). A pair‐wise comparison between rounds for each treatment showed significant differences in strike speed for the control group in R1 when compared with R2 (*p* = 0.0007), and R1 when compared with R3 (*p* < 0.0001), with the slowest strike speed found in R1. When comparing between treatments, differences in strike speed were also observed in each round. In R1, strike speed did not differ between treatments. In R2, fish in the control group displayed a faster strike speed in comparison to fish in the sedimentation treatment (*p* = 0.0006) and the multistressor treatment (*p* < 0.0001). Similarly, fish in the heat stress treatment also had a faster strike speed in comparison to fish in the sedimentation treatment (*p* = 0.006) and the multistressor treatment (*p* = 0.038), with no significant differences observed compared to the control group. In R3, *F. lapillum* in the control group exhibited significantly higher strike speeds compared to the heat stress (*p* = 0.0106), sedimentation (*p* < 0.0001) and multistressor treatments (*p* = 0.0001).

**FIGURE 4:**
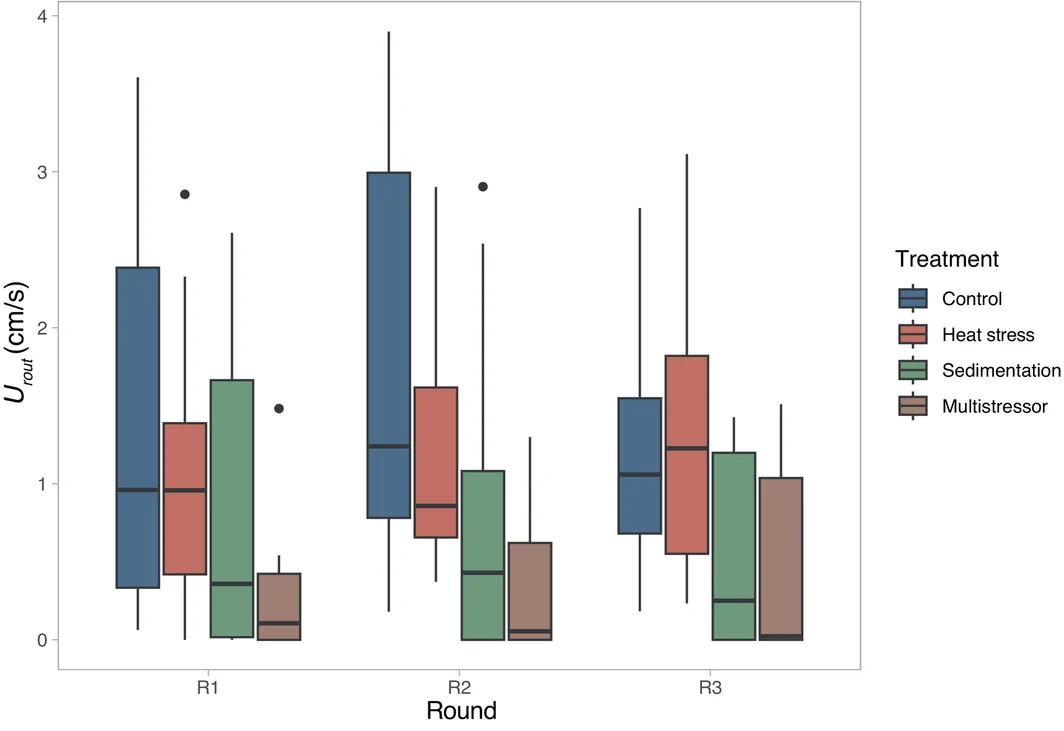
Box plots displaying *U*_*rout*_ of *F. lapillum* over three rounds of the experiment, comparing the four treatments.

*F. lapillum* routine swimming speed (*U*_*rout*_) changed significantly between treatments (ANOVA, F_[2.66]_ = 10.57, *p* < 0.0001), but no significant effect between treatments across rounds was found (Figure 4). Pair‐wise comparisons revealed that *U*_*rout*_ of fish in the control group differed significantly from those in both the sedimentation treatment (*p* = 0.0024) and the multistressor treatment (*p* < 0.0001), in which the routine swimming speed was at least 2 × faster for fish in the control treatment. In addition, fish in the heat stress treatment showed a significantly faster *U*_*rout*_ in comparison to the multistressor treatment (*p* = 0.0012).

**FIGURE 5:**
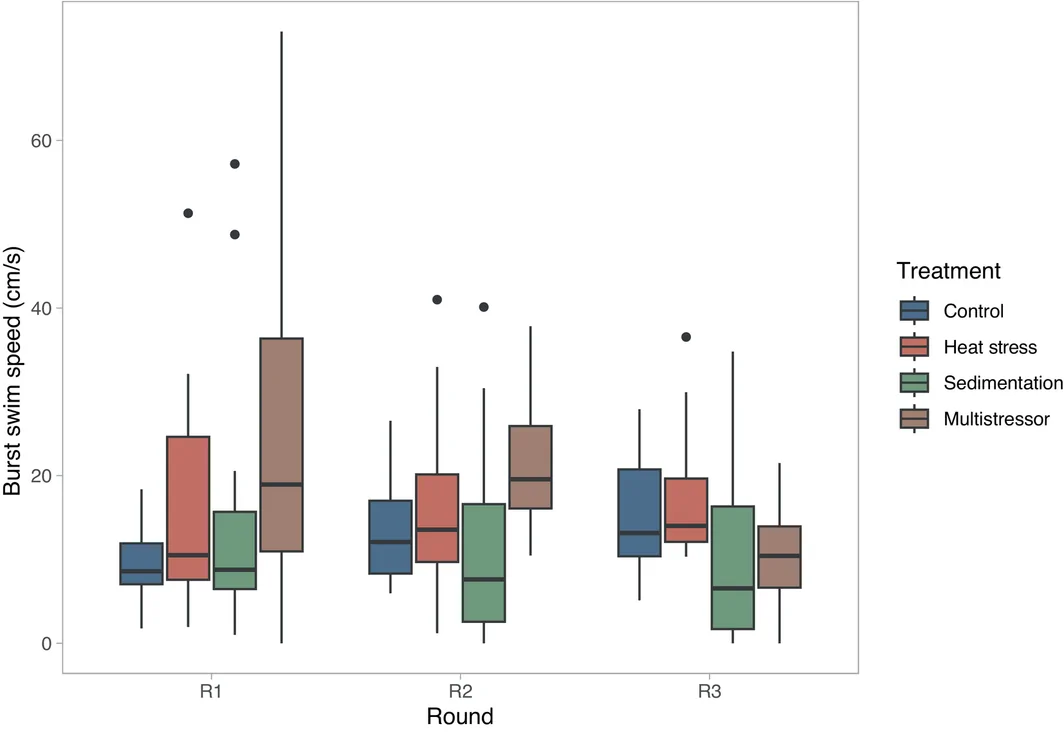
Box plots displaying the burst swim speed of *F. lapillum* over three rounds of the experiment, comparing the four treatments.

Burst swimming speed in *F. lapillum* did not significantly change between treatments across rounds (Figure 5).

**FIGURE 6:**
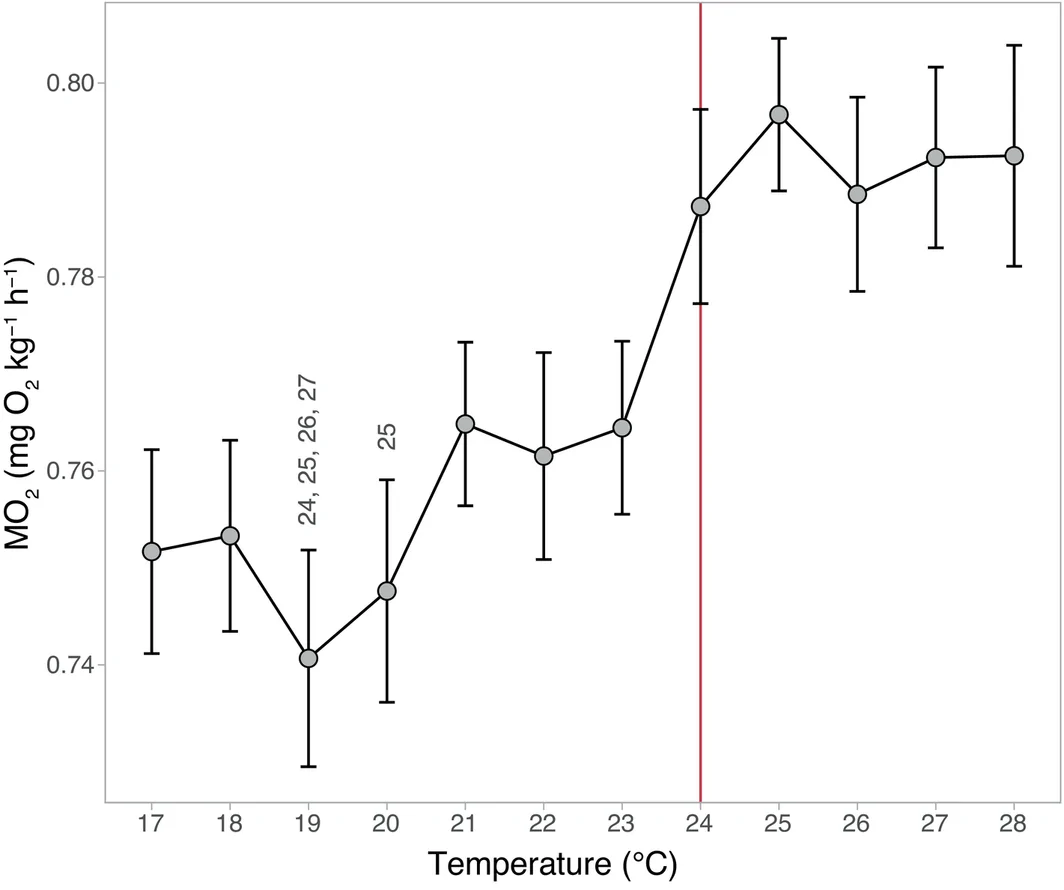
Mean oxygen consumption rate of *F. lapillum* (grey points) during acute thermal ramping from acclimation temperature until CTmax was reached at 28°C. Standard error bars are shown in black for each mean. Numbers above the means represent significant differences between temperatures, red line indicates a threshold where *MO*_*2*_ rate increases. All temperature values shown on the *x* axis may deviate by ±0.3°C.

CTmax for *F. lapillum* in this study was reached at 28°C, and their oxygen consumption rate increased significantly during acute thermal ramping trials (ANOVA, F_[1.82]_ = 3.95, *p* < 0.001). Pair‐wise comparisons between temperatures revealed significant differences in fish *MO*_*2*_ at 19°C when compared to fish at 24°C (*p* = 0.0355), 25°C (*p* = 0.0019), 26°C (*p* = 0.0265) and 27°C (*p* = 0.0138), with the highest *MO*_*2*_ rate found at temperatures above 24°C. Additionally, the *MO*_*2*_ rate at 20°C was significantly lower than at 25°C (*p* = 0.0137) (Figure 6).

## 4 DISCUSSION

Understanding fish performance responses to acute changes in environmental conditions, namely increases in temperature and turbidity, is essential for assessing the impacts of current and future climate change scenarios. In this present study, we found that in the first round of the experiment (R1), *F. lapillum* strike speed was the same in all treatments. However, in the subsequent two rounds, the strike speed of fish in the control group increased and was higher than all other treatments in R2 and R3. The initial lower values of *F. lapillum* strike speed could potentially be attributed to a possible chronic stress response from being moved from the acclimation tank to the experimental tanks, given that human handling and the threat of a change in housing condition can result in increased behavioural inhibition and latency to start feeding (Martins et al., 2012; Morgan & Tromborg, 2007; Resende, Streatfield, & Rogers, 2025).

A faster strike speed in subsequent trial rounds for *F. lapillum* in the control group could also be explained by feeding predictability or food anticipatory behaviour, which occurs when fish are expecting a positive stimulus from a conditioned schedule (e.g., addition of food to the tank) (Folkedal et al., 2012; Kleiber et al., 2024). Furthermore, the lack of an increase in strike speed for fish in the other treatments could be due to stress, given that fish experiencing stressful events are less sensitive to the positive stimulus of food, eliciting a decreased response in food anticipatory behaviour (Folkedal et al., 2012; Wendelaar Bonga, 1997).

In the second round of the experiment (R2), we found that the fish in the heat stress treatment struck faster than the individuals in the sedimentation treatment and in the multistressor treatment. Fish living in highly fluctuating environments tend to be able to adjust their behavioural and physiological responses in face of acute temperature increases (McArley et al., 2017; Resende et al., 2022; Stillman et al., 2025; Ziegler et al., 2023). Given that *F. lapillum* is an intertidal species subjected to frequent temperature fluctuation, we suggest that the temperature increase in R2 (i.e., 4°C greater than acclimation temperature) is still within the species’ optimal thermal range. Therefore, this increase did not result in a reduction in *F. lapillum* foraging performance.

*F. lapillum* that were subjected to increases in sedimentation, both in the individual sedimentation treatment and in the multistressor treatment, displayed a reduction in their strike speed in R2 and R3 relative to the control group. Turbidity can have a drastic impact on visual predators' ability to see prey in the water column, resulting in an increase in time to locate and effectively capture prey (Huenemann et al., 2012; Wing et al., 2021). In a study assessing the foraging efficiency of *Rhinecanthus aculeatus* under highly turbid waters, Newport et al. (2021) found that turbidity negatively affected foraging efficiency. Specifically, turbidity reduced the efficiency with which fish covered space to access prey, as measured by distance travelled and time taken to locate food. Similarly, in the present study, *F. lapillum*'s strike speed, which reflects time taken to capture food and efficiency in covering space, was negatively affected by increases in turbidity. This suggests that this species relies on visual cues for effective foraging. Another study by Johansen and Jones (2013) found that planktivorous coral reef fishes exhibited strong feeding sensitivity to sediment‐induced turbidity. Inshore species showed adaptation to local turbidity conditions, with foraging performance only reduced at levels exceeding those typically experienced in their habitats. Natural populations are often adapted to local environmental variability (Glockner Fagetti and Phillips, 2020). However, *F. lapillum* in the present study exhibited reduced foraging performance at turbidity levels that are commonly recorded in their local habitat (Supplementary Material Figure S2). This discrepancy may reflect that, in natural habitats, fish are exposed to short‐term sediment resuspension events and pulses of elevated turbidity (Cook et al., 2022; Resende et al., 2026), whereas individuals in this experiment experienced sustained exposure across successive rounds. Consequently, although *F. lapillum* may tolerate brief turbidity pulses in the wild, prolonged or recurrent exposure, as simulated in this experiment, may exceed their capacity to maintain optimal foraging performance. This suggests that under increasingly frequent sediment resuspension events, individuals may experience reduced feeding efficiency despite inhabiting naturally turbid environments.

When assessing the effects of suspended sediment concentration on *Acanthochromis polyacanthus* foraging behaviour, Wenger et al., 2012 found that fish reaction time to food input got progressively slower as sediment concentration increased. The authors discuss that the subsequent increases in water turbidity reduced the visual cues necessary for foraging and resulted in a delayed reaction time to food. Conversely, in the present study, the strike speed of *F. lapillum* in the sedimentation and multistressor treatments did not significantly differ from R2 to R3. This indicates that the second increase in turbidity and additional sediment input did not result in a slower reaction time to food. Therefore, the initial increase in turbidity from 0–10 NTU (R1) to 50–80 NTU (R2) is seems to be sufficient to impair the feeding performance of *F. lapillum*. *F. lapillum* is known to inhabit rock pools, which experience extreme thermal ramping and can reach >28°C on hot summer days (McArley et al., 2018). When examining the acute thermal ramping results in this study, it is noticeable that at ~24°C the mean oxygen consumption rate of *F. lapillum* starts increasing, which coincides with the temperature at which strike speed started to decrease in R3 of the performance assessment experiment relative to the control group. Moreover, in a study assessing *F. lapillum* MO_2_ rate responses to a simulated heatwave (22.5°C), Resende, Vinagre, & Rogers (2025b) found that fish respiration was not directly affected by increases in temperature. However, fish in the elevated temperature treatments lost weight, indicating that they were unable to meet their metabolic demands. These results indicate that oxygen delivery may be impaired at temperatures above 24°C, and that *F. lapillum* performance in natural environments could already be affected during extreme thermal ramping, even before oxygen consumption rate increases. This suggests that *F. lapillum* might experience thermal stress and reduced performance at temperatures well below CTmax (28°C) (McArley et al., 2017; McArley et al., 2018; Resende, Vinagre, & Rogers, 2025).

We acknowledge that only immobile prey were used in this study, which creates less contrast than moving prey and can be harder to detect amongst the sediment particles suspended in the water column (Johansen & Jones, 2013; Utne‐Palm, 2002). Utne‐Palm (2002) discusses that the negative effect of turbidity also depends on the distance at which the predator detects the prey and how large the prey is. Therefore, when interacting with evasive prey items, visual predators like *F. lapillum* can display an even greater reduction in foraging rate and attack success depending on prey size.

The swimming performance assessment in the present study reveals that the *U*_*rout*_ of *F. lapillum* did not differ between rounds; however, fish in the control group presented an overall higher *U*_*rout*_ compared to fish in the sedimentation and the multistressor treatment. Moreover, *F. lapillum* burst swimming speed did not significantly differ between treatments or rounds; hence, no combined effects of increasing water temperature and turbidity (i.e., multistressor treatment) were found in *F. lapillum* swimming performance.

Previous studies show that turbidity does not necessarily trigger a unique response in the swimming ability of fish; some fish species have been found to increase their movement, while others may reduce it or show no change in swimming activity (Gray et al., 2014; Rodrigues et al., 2023). Studies with guppies (*Poecilia reticulata*), a species that also inhabits highly fluctuating environments, revealed that fish are less active under high turbidity, potentially making them more vulnerable to predation (Allibhai et al., 2023; Kimbell & Morrell, 2015). *F. lapillum* are common prey for many other fish around New Zealand (Willis and Anderson, 2003), and we suggest that a reduction in swimming performance due to increased turbidity can either: (a) compromise fish escape response and increase risk of predation or (b) offer reduced predation risk from visual predators, given that the ability of predators to detect prey can be impaired by turbidity (Johansen & Jones, 2013; Newport et al., 2021; Shoup & Wahl, 2009). Therefore, since in this study *F. lapillum* burst swimming speed was maintained during increases in turbidity, we suggest that fish might face a lower predation risk in turbid waters, given that maintaining burst swimming speed helps minimize encounters with predators (da Silva et al., 2019; Walker et al., 2005).

In the present study, *F. lapillum* maintained burst swimming speed under acute increases in temperature, indicating that this escape performance trait was preserved across the thermal range tested. Similar patterns have been reported in the intertidal goby *Bathygobius cocosensis*, where burst swimming performance is maintained across temperature variations, suggesting a capacity to preserve rapid escape responses under thermal stress (da Silva et al., 2019). This functional stability is likely important for survival, as reduced escape speed can lower the probability of evading predators (Walker et al., 2005).

## 5 CONCLUSION

To our knowledge, this study provides the first evidence that *F. lapillum* are visual feeders, given that our results indicate that increases in turbidity through sediment suspension have a negative impact on *F. lapillum* feeding performance, as seen by the slower strike speed found in the sedimentation treatment. Additionally, even though *F. lapillum* burst swimming speed was not altered in any of the treatments, the individual and combined effects of heat stress and increased sediment concentration had an effect on *F. lapillum* swimming performance, as seen by the slower *U*_*rout*_ found in the sedimentation and the multistressor treatment. Moreover, the increase in oxygen consumption during acute ramping suggests rising metabolic demand at temperatures above 24°C, which may be associated with emerging constraints on performance. Future studies assessing how more severe increases in temperature and turbidity will affect *F. lapillum* performance are recommended, given that extreme thermal events are becoming more frequent and intense (Frölicher et al. 2018) and will greatly impact intertidal species behaviour and survival. The present study is the first to assess combined and individual effects of short‐term increases of turbidity and temperature on *F. lapillum* feeding and swimming behaviour; therefore, other possible behavioural adjustments for this species regarding other stressors remain unknown. Future research on the impacts of multiple stressors should also incorporate interspecific interactions, such as assessing *F. lapillum* predator escape response to understand how predator‐prey interactions could occur in natural environments. Moreover, future studies should evaluate how the feeding performance of *F. lapillum* responds to live, mobile prey and variation in prey size, enabling a more comprehensive assessment of potential effects across the trophic web.

## AUTHOR CONTRIBUTIONS

**Anna Carolina Resende**: Conceptualization (lead); writing—original draft (lead); formal analysis (lead); writing—review and editing (equal); Methodology (equal). **Alice Rogers**: Conceptualization (supporting); writing—review and editing (equal). **Catarina Vinagre**: Methodology (supporting); writing—review and editing (equal). Lucy Campbell: Methodology (equal); Investigation (lead).

## FUNDING INFORMATION

Catarina Vinagre acknowledges funding from UIDB/04326/2020, UIDP/04326/2020 and LA/P/0101/2020.

## Supporting information


**Figure S1.** Temporal variation in ocean temperature from April 2023 to March 2024 at sites in Wellington Harbour and the Wellington South Coast. Values represent weekly averages derived from hourly measurements at ~2 m depth.
**Figure S2.** Temporal variation in turbidity from April 2023 to March 2024 at sites in Wellington Harbour and the Wellington South Coast. Values represent biweekly averages from point measurements at ~1.2 m depth.
**Table S1.** Respiration (MO_2_) data from preliminary trials assessing the effect of acclimation duration (80, 40 and 20 min) on oxygen consumption in *F. lapillum*. No significant differences were detected among treatments, and a 20 min acclimation time was selected for subsequent experiments.

## Data Availability

The data that support the findings of this study are available from the corresponding author upon reasonable request.
