# Supplementary material for: Short‐term performance responses of an intertidal fish to sedimentation and warming
Source: J Fish Biol. 2026 May 8;109(2):1005–17. doi: 10.1111/jfb.70494 (PMC13521731; doi:10.1111/jfb.70494)
Supplement: Supplementary file 1 [file JFB-109-1005-s001.docx]

**Figure S1 –** Ocean temperature variation from April 2023 to March 2024 around the Wellington Harbour and the Wellington South Coast. Lines connect weekly averaged values, calculated from hourly measurements. Temperature was measured with HOBO TidbiT v2UTBI-001 loggers at a ~2 m depth in 3 sites in the harbour and 3 sites in the south coast.

**Figure S2 –** Turbidity variation from April 2023 to March 2024 around the Wellington Harbour and the Wellington South Coast. Lines connect biweekly averaged values from point measurements. Turbidity was measured with RBRconcerto³ CTD at a ~1.2 m depth in 3 sites in the harbour and 3 sites in the south coast.

**Table S1** - Respiration data from preliminary trials testing different acclimation times of *F. lapillum*. Preliminary trials were conducted to determine the optimal acclimation time prior to measuring oxygen consumption (MO₂). Three acclimation durations were tested (T1 = 80 min, T2 = 40 min, and T3 = 20 min), each with six individual fish (n = 6 per treatment). Following each acclimation period, MO₂ was measured using the same protocol applied in the main experiment—over a one-hour period, with a 5-minute flush every 15 minutes, resulting in three MO₂ measurements per fish. A one-way ANOVA was performed to assess differences in MO₂ across the three acclimation durations, and no significant differences were detected. Based on these results and to optimize logistical efficiency, the shortest acclimation time (20 minutes) was selected for the experimental trials.

| time | fishid | mo2cor |
| --- | --- | --- |
| T1 | 1 | 0.79173961 |
| T1 | 2 | 0.76726884 |
| T1 | 3 | 0.75222165 |
| T1 | 4 | 0.78002292 |
| T1 | 5 | 0.84189096 |
| T1 | 6 | 0.75245262 |
| T2 | 1 | 0.70257467 |
| T2 | 2 | 0.7629519 |
| T2 | 3 | 0.76314202 |
| T2 | 4 | 0.79156481 |
| T2 | 5 | 0.77645072 |
| T2 | 6 | 0.75245262 |
| T3 | 1 | 0.69029152 |
| T3 | 2 | 0.78400068 |
| T3 | 3 | 0.64862383 |
| T3 | 4 | 0.77103491 |
| T3 | 5 | 0.79495814 |
| T3 | 6 | 0.75729101 |
